# Unveiling the Influences of In Situ Carbon Content on the Structure and Electrochemical Properties of MoS_2_/C Composites

**DOI:** 10.3390/molecules29184513

**Published:** 2024-09-23

**Authors:** Bofeng Zhang, Junyao Zhao, He Zhang, Jian Tian, Yang Cui, Wenjun Zhu

**Affiliations:** 1 School of Mechanical and Electrical Engineering, Jingdezhen Ceramic University, Jingdezhen 333403, China; 2 School of Materials Science and Engineering, Zhejiang University, 38 Zheda Road, Xihu District, Hangzhou 310027, China; 3 School of Materials Science and Engineering, College of Chemical and Biological Engineering, Shandong University of Science and Technology, Qingdao 266590, China; 4 Ceramic Research Institute of Light Industry of China, Jingdezhen 333000, China

**Keywords:** MoS_2_, carbon, heterostructure, in situ synthesis, lithium–ion batteries

## Abstract

In this work, a MoS_2_/C heterostructure was designed and prepared through an in situ composite method. The introduction of carbon during the synthesis process altered the morphology and size of MoS_2_, resulting in a reduction in the size of the flower-like structures. Further, by varying the carbon content, a series of characterization methods were employed to study the structure and electrochemical lithium storage performance of the composites, revealing the effect of carbon content on the morphology, structure characteristics, and electrochemical performance of MoS_2_/C composites. The experimental setup included three sample groups: MCS, MCM, and MCL, with glucose additions of 0.24 g, 0.48 g, and 0.96 g, respectively. With increasing carbon content, the size of MoS_2_ initially decreases, then increases. Among these, the MCM sample exhibits the optimal structure, characterized by smaller MoS_2_ dimensions with less variation. The electrochemical results showed that MCM exhibited excellent electrochemical lithium storage performance, with reversible specific capacities of 956.8, 767.4, 646.1, and 561.4 mAh/g after 10 cycles at 100, 200, 500, and 1000 mA/g, respectively.

## 1. Introduction

As global technology progresses and environmental challenges intensify, the need for efficient energy storage solutions has become increasingly urgent. Lithium–ion batteries (LIBs), as the most mature and widely applied battery technologies today, are considered crucial for advancing electric vehicles, integrating renewable energy sources, and developing portable electronic devices [1,2,3]. However, with rising performance demands, traditional graphite anode materials have revealed several shortcomings, including a relatively low theoretical capacity (372 mAh/g), slow ion-diffusion rate, and suboptimal rate performance [4,5,6]. These limitations restrict the further development of LIBs for applications requiring high energy density and fast charging/discharging capabilities [7,8,9]. Consequently, there is a growing focus on exploring promising alternative carbon materials to address these limitations and enhance battery performance.

MoS_2_, as a typical two-dimensional layered material, has a graphene-like structure consisting of three atomic layers of S-Mo-S stacked together [10,11,12]. The weak van der Waals forces between the layers facilitate easier shuttling of Li^+^ ions. With a theoretical specific capacity of 670 mAh/g, it is significantly higher than that of graphite materials [13,14,15]. LIBs can benefit from MoS_2_, which is, thus, considered a promising anode material. However, the substantial changes in volume during charge and discharge cycles, along with the intrinsic low electrical conductivity, severely restrict the advancement of MoS_2_ [16,17]. To address these challenges, researchers have developed two primary strategies. The first strategy involves improving the structural design of MoS_2_ to maintain its structural integrity [18]. This can be achieved by fabricating MoS_2_ with various morphologies, such as nanosheets and nanospheres, to increase interlayer spacing and surface area, thereby providing more active sites for electrochemical reactions [19,20,21,22]. The second strategy is to construct heterostructure composites by combining MoS_2_ with conductive materials to enhance its electrical conductivity [23]. Carbon materials, due to their excellent conductivity and abundant resource, have been widely applied in this field. Numerous MoS_2_/C-based composites have been reported, demonstrating improved electrochemical performance [24,25,26,27]. As is well known, the electrochemical properties are greatly influenced by the structure; at the same time, the structure is affected by the composition and content of their components [28,29]. Zhong et al. [8] reported that the graphene content significantly affects the electrochemical performance and structure of composite materials. When the mass ratio of MoS_2_ to graphene is 1:1, performance is optimal, achieving a specific capacity of 664 mAh/g after 300 cycles at 250 mA/g. Despite these insights, there remains a gap in understanding how varying carbon content affects the structure and performance of MoS_2_/C composites. Further research is needed to elucidate the relationship between carbon content, microstructure, morphology, and electrochemical performance in MoS_2_/C composites.

**Figure 1:**
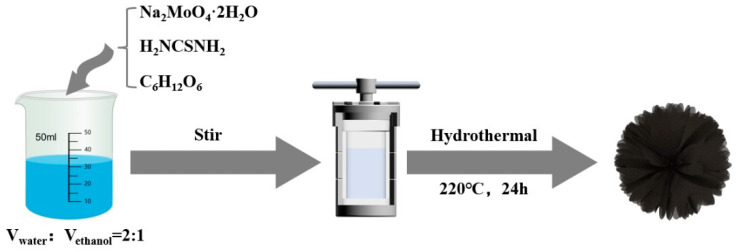
The synthetic process of MoS_2_/C composite.

In this study, MoS_2_/C composites were synthesized using a simple one-step hydrothermal method (Figure 1), and the effects of varying glucose content on the morphology, structure, and electrochemical performance of the composites were systematically investigated. The results show that the morphology and microstructure of composites are significantly affected by the content of the carbon component, and the optimal structure is obtained for MCM with the addition of 0.48 g of glucose. Meanwhile, benefitting from the in situ-generated carbon, the stable heterogeneous is formed between MoS_2_ and carbon. This unique structural configuration endows the composite with enhanced structural stability and superior electrochemical performance.

## 2. Results and Discussion

**Figure 2:**
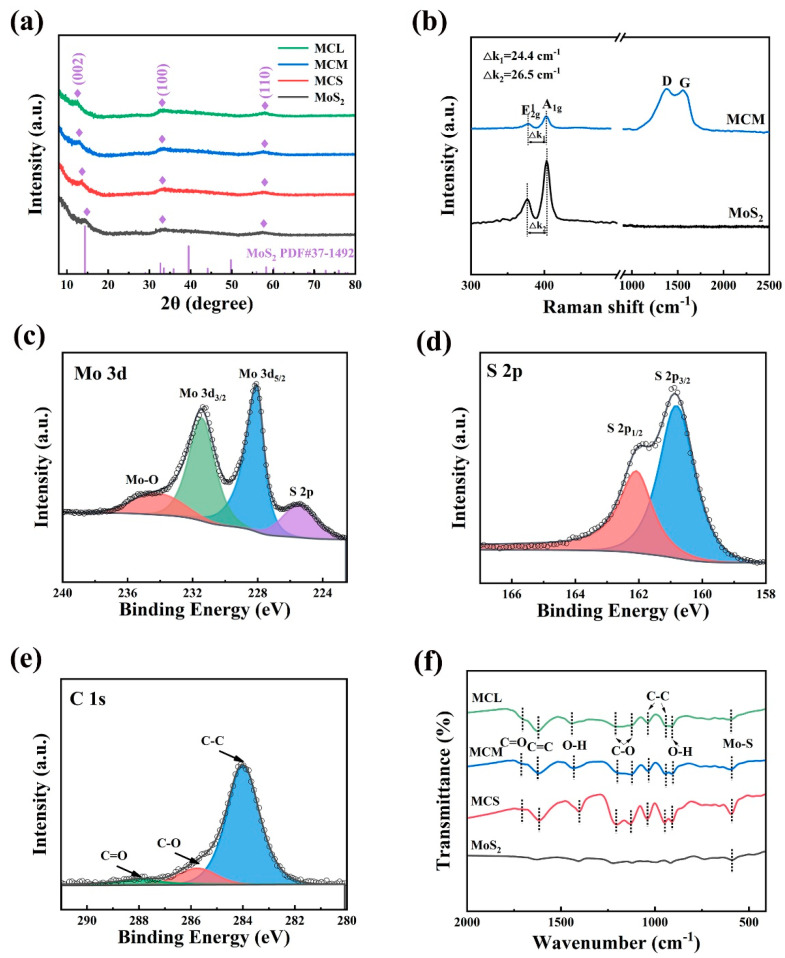
(**a**) XRD results of all the samples, (**b**) Raman spectra, (**c**–**e**) XPS results of MCM composite, (**f**) FT-IR spectra of all the samples.

As shown in Figure 2a, it can be found that the diffraction patterns of all the samples (MoS_2_ and MoS_2_/C composites) present similar characteristics, which are attributed to hexagonal 2H-MoS_2_ (JCPDS 37-1492), with distinct peaks observed at 32.8° and 58.2°, corresponding to the (100) and (110) planes, respectively [30]. Notably, the peaks corresponding to the (002) plane shift progressively to lower angles with increasing carbon content, observed at 14.3°, 13.6°, 13.1°, and 12.5°. Additionally, compared to pure MoS_2_, the corresponding peak intensities of the MoS_2_/C composites show a slight decrease. This is because the in situ-formed carbon derived from glucose in the samples affects the crystallinity of MoS_2_, resulting in reduced peak intensity in the XRD patterns when crystallinity is low [16]. According to Bragg’s equation 2dsinθ = nλ, the interlayer spacings at the (002) plane are calculated to be 0.61, 0.65, 0.67, and 0.69 nm, respectively. This indicates that the insertion of carbon leads to an increase in the interlayer spacing of MoS_2_, which facilitates Li^+^ deintercalation and intercalation and improves the structure stability [31]. No obvious impurity peaks were detected, indicating the high purity characteristic of the prepared samples. As shown in Figure 2b, both pure MoS_2_ and MCM exhibit similar spectral patterns in the first half of the spectrum. Specifically, the peak at 378.0 cm^−1^ in MCM corresponds to the $E_{2g}^{1}$ mode, which is associated with the in-plane bending of sulfur and molybdenum atoms, while the peak at 402.5 cm^−1^ is attributed to the A_1g_ mode, related to the out-of-plane vibration of sulfur atoms [32]. However, the energy difference k_1_ of MCM (24.4 cm^−1^) is significantly lower than k_2_ for pure MoS_2_ (26.5 cm^−1^), indicating that the MoS_2_ crystals in MCM are primarily composed of few-layer MoS_2_ nanosheets, suggesting that the addition of carbon has alleviated the stacking issue [33,34,35,36,37]. Additionally, for MCM, D, and G bands are detected at 1363.5 and 1568.3 cm^−1^, respectively. The D peak represents lattice defects in carbon, such as N-doping or vacancies, while the G peak is associated with sp^2^-hybridized carbon. As shown in Figure S1a, the high intensity ratio of the D and G peaks (I_D_/I_G_ = 1.21) indicates the presence of amorphous carbon [38,39,40]. In addition, Figure S1b shows the Raman spectrum in the 100–500 cm^−1^ range, and no Mo-O bonds are observed. Figure S2 shows the full XPS survey spectrum of MCM, revealing the presence of O, C, Mo, and S elements within the sample, with their respective contents indicated in the inset. The Mo-to-S atomic ratio is 2.05, which is in close agreement with the stoichiometric ratio [41]. In addition, a significant amount of O was detected in the full spectrum. However, no Mo-O bonds are observed in the Raman and FT-IR, indicating that the oxygen originates from subsequent processing rather than the initial synthesis. In the Mo 3d spectrum (Figure 2c), four distinct peaks at 234.2, 231.5, 228.1, and 225.4 eV are observed, which correspond to Mo-O, Mo^4+^ 3d_3/2_, Mo^4+^ 3d_5/2_, and S 2s, respectively [42,43]. Two characteristic peaks at 162.1 and 160.8 eV in the S 2p spectrum (Figure 2d) correspond to the spin-orbit doublet states of S 2p_1/2_ and S 2p_3/2_ in MoS_2_ [44]. The C 1s spectrum (Figure 2e) exhibits peaks at 288.1, 285.5, and 284.1 eV, corresponding to C=O, C-O, and C-C bonds, respectively, indicating the formation of in situ-generated carbon material [45,46]. According to the above results, the heterostructure composed of carbon and MoS_2_ is proved. The composition of the MoS_2_/C materials was further confirmed by FT-IR spectra. As shown in Figure 2f, despite the functional group signals in the MoS_2_ sample being relatively weak, the Mo-S bond peak can still be clearly detected. Besides the Mo-S bond, the pronounced peak (1623.1 cm^−1^) of the C=C bond is found in MoS_2_/C composites, indicating the existence of carbon and MoS_2_, and similar characteristic peaks can be found for all the MoS_2_/C composites [47,48,49]. In addition, the C=O, C-O, and C-C bonds are also observed in the FT-IR spectrum, which is highly consistent with the XPS results [50].

**Figure 3:**
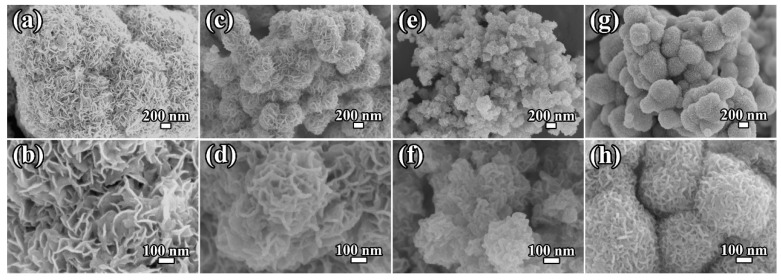
SEM images of the (**a**,**b**) MoS_2_, (**c**,**d**) MCS, (**e**,**f)** MCM, and (**g**,**h**) MCL.

To observe and analyze the morphology of the samples, SEM was employed. As shown in Figure 3, a flower-like microsphere morphology can be detected from all samples. For pure MoS_2_, the overall size of the flower-like microspheres is about 2 μm (Figure 3a), and the microspheres are self-assembled from smooth nanosheets about 150 nm in size. In contrast, the MoS_2_/C composites show somewhat suppressed growth of MoS₂ due to the in situ-generated carbon, resulting in the smaller microsphere sizes (Figure 3c,e,g). As shown in the SEM results, the diameters of MoS_2_ in MCS, MCM, and MCL are 250–450 nm, 150–250 nm, and 200–600 nm, respectively. It can be found that the flower-like structure exhibits a smaller and more uniform size distribution in MCM. High-resolution SEM images (Figure 3d,f,h) further illustrate that the nanosheets in the MoS_2_/C composites exhibit a more curled morphology and increased thickness compared to pure MoS_2_. As the glucose content increases from 0.24 g to 0.48 g, the size of the microspheres decreases, and the nanosheets in the MCM sample display greater thickness (Figure 3e). When the glucose content is further increased to 0.96 g, the nanosheets in the MCL sample exhibit the smallest size and largest thickness, with noticeable agglomeration (Figure 3g,h). This agglomeration is attributed to the increased amount of in situ-generated carbon. Based on this observation, it can be inferred that MCM likely possesses the largest specific surface area, followed by MCS and MCL, with pure MoS_2_ having the smallest surface area. A larger specific surface area facilitates the transfer of ions and electrons, which enhances Li storage and leads to superior electrochemical performance.

**Figure 4:**
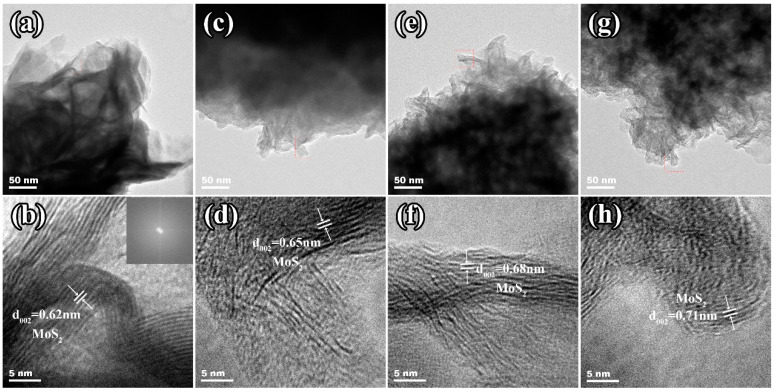
The TEM images of the (**a**) pure MoS_2_, (**c**) MCS, (**e**) MCM, and (**g**) MCL and HRTEM images of the (**b**) pure MoS_2_ (inset is the SAED image), (**d**) MCS, (**f**) MCM, and (**h**) MCL.

Figure 4 presents the TEM images used to investigate the microstructural characteristics of the samples. The flower-like structure observed in all samples from the TEM images (Figure 4a,c,e,g) aligns with the SEM results (Figure 3). The clear lattice fringes with an interlayer spacing of 0.62 nm in Figure 4b are attributed to the (002) plane of 2H-MoS_2_ [51]. Additionally, the SAED result (inset of Figure 4b) confirms the polycrystalline nature of MoS_2_. The MoS_2_/C materials exhibit larger interlayer spacing compared to pure MoS_2_, which increases gradually with increasing carbon content, from 0.65 nm for MCS (Figure 4d) to 0.68 nm for MCM (Figure 4f) and 0.70 nm for MCL (Figure 4h). Additionally, the lattice fringes of the MoS_2_/C composites are more blurred compared to those of pure MoS_2_, indicating a higher degree of structural disorder [52]. This observation is further supported by the XRD results, which show that the MoS_2_/C composites have lower crystallinity compared to pure MoS_2_.This is because the formation of MoS_2_ is accompanied by the in situ generation of disordered carbon derived from the reduction in glucose. The in situ-formed disordered carbon coats the MoS_2_ nanosheets and is embedded between the MoS_2_ layers, partially inhibiting the growth of MoS_2_. This results in the formation of MoS_2_/C composites with smaller sizes, larger interlayer spacing, and lower crystallinity [53,54].

**Figure 5:**
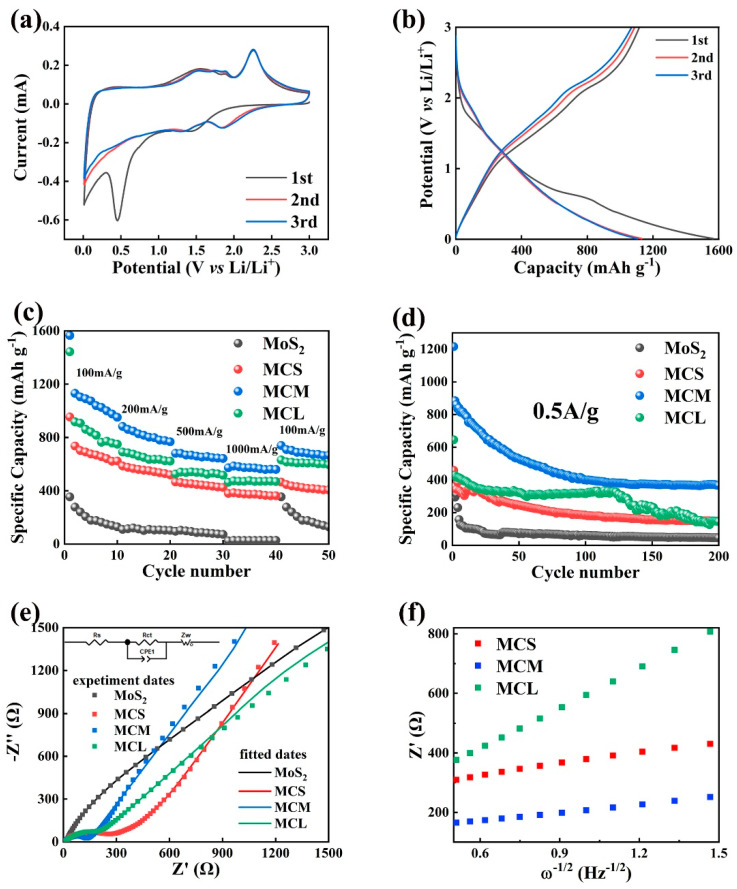
(**a**) CV curves at 0.01 mV s^−1^ and (**b**) charge–discharge profiles of the MCM, (**c**) rate curve, (**d**) circulation curve at 0.5 A/g, (**e**) Nyquist plots of the pure MoS_2_ and MoS_2_/C, (**f**) Z’–w^−1/2^ curves at low frequency of MoS_2_/C composites.

Figure 5a shows the CV results. Two distinct peaks were observed during the first cathodic scan at 1.45 V and 0.44 V, which are attributed to the formation of Li_x_MoS_2_ and the further lithium insertion process (MoS_2_ + 4Li^+^ + 4e^−^→Li_x_MoS_2_, Li_x_MoS_2_ + (4 − x)Li^+^ + (4 − x)e^−^→Mo + 2Li_2_S) as well as the formation of the SEI layer [55]. The oxidation peak at 1.56 V during the initial anodic scan corresponds to the partial oxidation of Mo; while around 2.26 V, another peak is observed, which relates to the oxidation of Li_2_S to S [56]. In subsequent cycles, the oxidation peak position remained unchanged, while the reduction peaks are replaced by two peaks at 1.84 and 1.33 V, resulting from the conversion processes of MoS_2_ to Li_x_MoS_2_ and S to Li_2_S, respectively [57].The good reversibility of the electrode is evidenced by the nearly overlapping curves observed in the second and third cycles. During the charge–discharge process, similar CV results are detected from pure MoS_2_, MCS, and MCL composites, as shown in Figure S3, indicating comparable electrochemical reactions.

Figure 5b displays the charge–discharge curves of the MCM composite at 100 mA/g for the first three cycles. The voltage plateaus observed are consistent with the CV results, and the curves overlap well after the second cycle, demonstrating stable cycling behavior. The Coulombic efficiency increased from 71.2% to 96.8%, indicating good reversibility of the material. Moreover, compared to the other samples (Figure S4), the MCM exhibits superior performance with discharge capacities of 1565.7, 1132.1, and 1101.8 mA h/g in the first three cycles, respectively. Figure 5c shows the rate performance of pure MoS_2_ and MoS_2_/C composites. Evidently, owing to the heterogeneous composite structure, the MoS_2_/C composites present superior rate performance in comparison to pure MoS_2_. Additionally, among the composite materials, MCM exhibits the highest reversible capacity, with reversible specific capacities of 956.8, 767.4, 646.1, and 561.4 mAh/g after 10 cycles at 100, 200, 500, and 1000 mA/g, respectively. After 200 cycles, Figure 5d shows that the reversible specific capacities of pure MoS_2_, MCS, MCM, and MCL are 47.8, 152.3, 367.0, and 146.7 mAh/g, respectively. The results demonstrate that the enhanced electrochemical lithium storage performance arises from the effective integration of carbon with molybdenum disulfide; moreover, with an appropriate amount of carbon, the MCM composite displays the superior cycle stability and rate performance. The capacity decline of MCM during cycling may be related to changes in electrolyte concentration and the presence of byproducts from electrochemical reactions [58,59].

To further understand the electrochemical performance of MoS_2_/C electrodes, EIS tests were performed. Figure 5e presents the EIS spectra of MoS_2_/C composites; the inset is the equivalent circuit used for fitting. The R_ct_ values for MoS_2_/C composites (MCS, MCM, and MCL) are 192, 134.1, and 174.1 Ω, respectively. Clearly, the R_ct_ value of pure MoS_2_ (>>1000 Ω) is much higher than that of MoS_2_/C composites, indicating that the addition of carbon significantly enhances the conductivity of the composites [11]. Among the composites, the MCM shows the lowest R_ct_ value, which can be attributed to its optimal structural configuration and appropriate carbon content. Figure 5f shows the linear portion of the EIS curves at low frequencies (fitted using the equation Z′ = R_s_ + R_ct_ + σω^−1/2^) to calculate the lithium–ion diffusion coefficient (D_Li_^+^). The D_Li_^+^ for MCS, MCM, and MCL are calculated to be 6.64 × 10^−16^, 1.73 × 10^−15^, and 7.08 × 10^−17^, respectively, using the formula *D*_Li_^+^ = *R*^2^*T*^2^*/*2*n*^4^*F*^4^*C*^2^*σ*^2^*A*^2^ (where T is the temperature, F is the Faraday constant, R is the gas constant, *n* is the number of electrons per molecule during oxidation, σ is the slope from Figure 5f, A is the surface area of the active electrode, and C is the concentration of Li^+^) [60]. MCM has the highest D_Li_^+^, which is attributed to its optimal structure configuration and enhanced electron transport performance contributed by the in situ-formed carbon [61].

**Figure 6:**
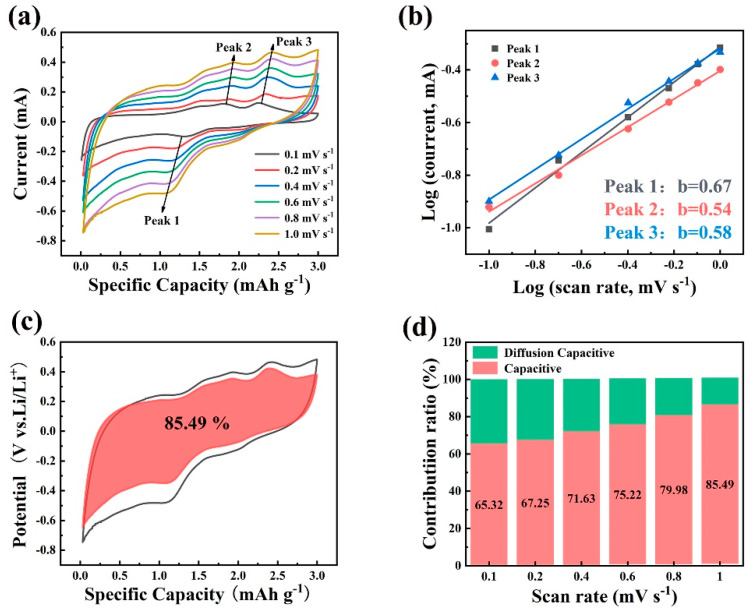
(**a**) CVs of MCM electrode, (**b**) fitting line of log (v, mV/s) −log (I_peak_, mA), (**c**) capacitive contribution at 1.0 mV·s^−1^, (**d**) ratios of pseudocapacitive and diffusion-controlled contributions at different sweep rates.

To explore the electrochemical kinetics of the MCM composite, CV tests were performed across scan rates ranging from 0.1 to 1 mV/s. Figure 6a demonstrates that as the scan rate increases, the shape of the CV curves for MCM remains consistent, highlighting the excellent electrochemical reversibility [13]. The b values were obtained using the power-law equation (*i = av^b^*). When the b value approaches 1 or 0.5, it indicates that the reaction kinetics are primarily capacitive-controlled or diffusion-controlled, respectively [62]. The corresponding b values for peaks 0.67, 0.54, and 0.58 in Figure 6b indicate that capacitive control predominates in the electrochemical kinetics of the MCM electrode. Using the equation *i(V) = k*_1_*v + k*_2_*v*^1/2^, the ratio of diffusion and capacitive contributions at different scan rates can be quantified [4]. At a scan rate of 1.0 mV s^−1^, Figure 6c demonstrates that the capacitive contribution of the MCM electrode amounts to 85.49%, significantly higher than that of pure MoS_2_ (Figure S5), indicating the superior structural stability and electrochemical lithium storage performance. Figure 6d shows that as the scan rate increases, the percentage of capacitive contribution increases from 65.32% to 85.49%, indicating that capacitive control is the dominant reaction behavior during lithium storage, further demonstrating the excellent electrochemical performance of the MCM composite electrode. Table 1 compares the performance of our work to that of existing MoS_2_-based composites. It can be observed that the MCM in this study exhibits comparable electrochemical performance. The results can be explained by the following factors: Firstly, the electric conductivity of the composite can be improved by the in situ-formed carbon derived from glucose. Secondly, an appropriate carbon content ensures that optimal structure and structural stability, such as the in situ-generated carbon shell, can effectively adsorb and solidify the Mo and Li_2_S generated in the electrochemical reaction, the structural collapse can be restrained, and the enhanced structural stability can be obtained. Moreover, owing to the synergistic effect of a heterogeneous structure combined with MoS_2_ and carbon, this results in excellent electrochemical reaction kinetics.

## 3. Experimental Section

### 3.1. Materials Synthesis

All reagents used in this study were of analytical grade and required no further purification. The synthesis steps for MoS_2_/C composites are as follows: First, 1.5 mmol of sodium molybdate dihydrate (Na_2_MoO_4_·2H_2_O, Tianjin Damo Chemical Reagent Factory, China, AR analytical purity) and 6 mmol of thiourea (H_2_NCSNH_2_, Tianjin Fengchuan Chemical Technology Co., Ltd., Tianjin, China, AR analytical purity) were added to a mixed solution of 20 mL deionized water and 10 mL ethanol. After stirring the mixed solution for 30 min, 0.48 g of glucose (C_6_H_12_O_6_, Xilong Science Co., Ltd., Shantou, China, AR analytical purity) was added and stirred until completely dissolved. The resulting solution was then transferred to a closed reaction vessel and reacted at 220 °C for 24 h. After natural cooling, the sample was washed three times with ethanol and water, followed by drying in an oven, which was denoted as MCM. In addition, with a consistent reaction condition except for the glucose content changing to 0.24 and 0.96 g, the final products were denoted as MCS and MCL, respectively. Moreover, pure MoS_2_ was also synthesized using identical methods, excluding the addition of glucose.

### 3.2. Material Characterization

The composition of the materials was analyzed using a Philips X’Pert Pro instrument (XRD, λ = 0.15418 nm, Bruker AXS GmbH, Bellerica, MA, USA) and a Raman spectrometer (Thermo Fisher DXR Smart Raman, Renishaw, Shanghai, China, 532 nm laser source, in Regular mode, with a grating of 1800 L/mm (vis), the exposure time was 10 s, the laser power was set to 10%, and the scan range covered 0–3200 cm^−1^ Raman shift, using a 50× objective lens). The surface composition was analyzed using X-ray photoelectron spectroscopy (Thermo Scientific, Shanghai, China, XPS Thermo Scientific K-Alpha^+^, with an Al Kα radiation source (hv = 1486.6 eV)). All peak positions were calibrated relative to the C 1s peak at 284.80 eV. The morphology and structure of the samples were studied using SU-8100 model field emission scanning electron microscopy (SEM, HITACHII, Beijing, China) and JEM-2010 model transmission electron microscopy (TEM, JEOL, Beijing, China), operating at acceleration voltages of 5 kV and 200 kV, respectively.

### 3.3. Electrochemical Measurements

The battery assembly was conducted inside an Ar-filled glove box (MIKROUNA Super 900, Shanghai, China), where concentrations of water and oxygen were maintained below 0.1 ppm. Coin cells of CR2032 type (Keludi, Guangdong, China) were employed, comprising lithium foil as the electrode and a porous Celgard 2300 separator (Celgard, NC, USA). The electrolyte composition consists of 1 M LiPF_6_ mixed with ethylene carbonate (EC), ethyl methyl carbonate (EMC), and dimethyl carbonate (DMC) (V_EC_:V_EMC_:V_DMC_ = 1:1:1). The sample, conductive agent (acetylene black), and binder (polyvinylidene fluoride) were mixed in a mass ratio of 7:2:1 in N-methyl-2-pyrrolidone (NMP) to form a slurry, which was stirred uniformly for 4 h. Subsequently, the slurry was evenly coated onto copper foil with a diameter of 14 mm and dried overnight at 120 °C in a vacuum oven to form the anode electrode. The specific capacity in this study was determined based on the weight of the active material in the anode electrode, with each copper foil carrying approximately 1.2 mg of active material loading. The charge–discharge performance at different current densities was evaluated using the Shenzhen Neware BTS battery testing system (CT-4008, Neware, Shenzhen, China). Cyclic voltammetry (CV) experiments at a scan rate of 0.1 mV/s were conducted using a CHI660e electrochemical workstation (Chenhua, Shanghai, China) with a voltage range from 0.05 to 3.0 V. Meanwhile, electrochemical impedance spectra (EIS) were obtained on the same electrochemical workstation, with an amplitude of 5.0 mV and a scan frequency range from 100 kHz to 1 mHz. Additionally, EIS results were fitted using Zview 3.1.

## 4. Conclusions

In summary, MoS_2_/C composites were synthesized via a straightforward one-step hydrothermal method. The study systematically examined the impact of varying carbon content on the structure and electrochemical performance of these composites. The findings reveal that an optimal carbon content leads to the formation of an ideal composite (MCM), characterized by a well-defined heterostructure between MoS_2_ and carbon and uniformly distributed flower-like microspheres. As an anode for LIBs, MCM demonstrates excellent electrochemical performance, with a specific capacity of 314.9 mAh/g after 100 cycles at 0.5 A/g and a capacitive contribution of 85.49% at 1.0 mV/s. These results underscore the composite’s exceptional potential for future energy storage applications.

## Figures and Tables

**Table 1:** Comparison of electrochemical properties to existing MoS_2_-based composites.

Materials	Cyclic Performance (mAh g^−1^/A g^−1^)	Rate Performance (mAh g^−1^/A g^−1^)	Refs
MoS_2_ nanoflakes	530/0.1 (after 100 cycles)	1080/0.1, 260/1, 400/0.1	[12]
MoS_2_@Mo_2_C	145/0.05 (after 100 cycles)	210/0.01, 89/0.2, 210/0.01	[63]
MoS_2_@SnO_2_	277/0.1 (after 100 cycles)	600/0.01, 290/0.1, 510/0.01	[64]
MoS_2_/C	790/0.1 (after 50 cycles)	854.3/0.1, 140.9/3, 734.2/0.1	[6]
MCM	411.7/0.5 (after 100 cycles)	1124/0.1, 585/1, 742/0.1	This work

## Data Availability

The raw data supporting the conclusions of this article will be made available by the authors on request.

## Supplementary Materials

The following supporting information can be downloaded at: https://www.mdpi.com/article/10.3390/molecules29184513/s1, Figure S1: The enlarged Raman spectra in range of (a) 1000–2000 cm^−1^ and (b) 100–500 cm^−1^, respectively. Figure S2: Survey spectra of MCM composite, insets show the actual content of O, C, Mo and S elements. Figure S3: CV curves at 0.01 mV s^−1^ of pure MoS_2_, MCS and MCL. Figure S4: Charge-discharge profiles of pure MoS_2_, MCS and MCL. Figure S5: (a) CVs of pure MoS_2_ electrode (0.1–1.0 V·s^−1^), (b) Fitting line of log (v, mV/s)-log (I_peak_, mA), (c) Capacitive contribution at 1.0 mV·s^−1^, (d) Ratios of pseudocapacitive and diffusion controlled contributions at different sweep rates.
