# Supplementary material for: Unveiling the Influences of In Situ Carbon Content on the Structure and Electrochemical Properties of MoS2/C Composites
Source: Molecules. 2024 Sep 23;29(18):4513. doi: 10.3390/molecules29184513 (PMC11435134; doi:10.3390/molecules29184513)
Supplement: Supplementary file 1 [file molecules-29-04513-s001.zip › molecules-3125666-supplementary.pdf]

Supplementary Materials

Unveiling the Influences of In Situ Carbon Content on the Structure and Electrochemical Properties of MoS2/C Composites

Bofeng Zhang [1] , Junyao Zhao [1] , He Zhang [3] , Jian Tian [2,*], Yang Cui [4] and Wenjun Zhu [1,*]

1 School of Mechanical and Electrical Engineering, Jingdezhen Ceramic University, Jingdezhen 333403, China; 2220034002@stu.jcu.edu.cn (B.Z.); 1920036003@stu.jcu.edu.cn (J.Z.)
2 School of Materials Science and Engineering, College of Chemical and Biological Engineering, Shandong University of Science and Technology, Qingdao 266590, China
3 School of Materials Science and Engineering, Zhejiang University, 38 Zheda Road, Xihu District, Hangzhou 310027, China; zhhe@zju.edu.cn
4 Ceramic Research Institute of Light Industry of China, Jingdezhen 333000, China; 2220038006@stu.jcu.edu.cn
* Correspondence: jiantian@sdust.edu.cn (J.T.); zhuwj@zju.edu.cn or zwj2730038@126.com (W.Z.)

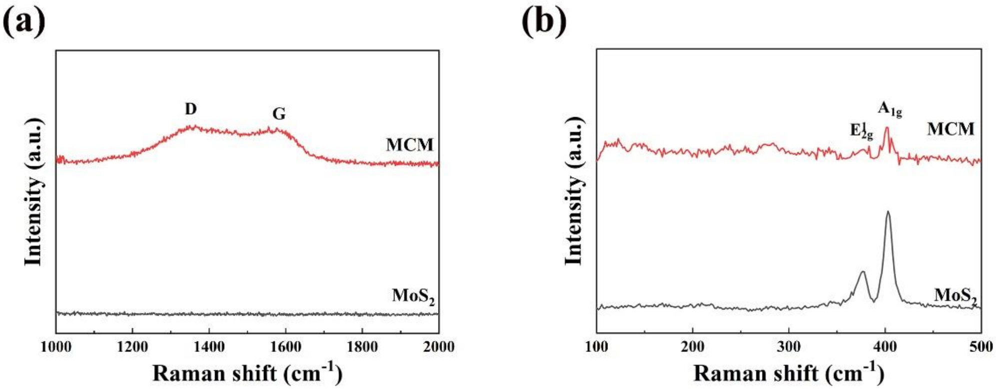

Figure S1. The enlarged Raman spectra in range of (a)1000-2000 cm^{-1} and (b) 100-500 cm^{-1}, respectively.

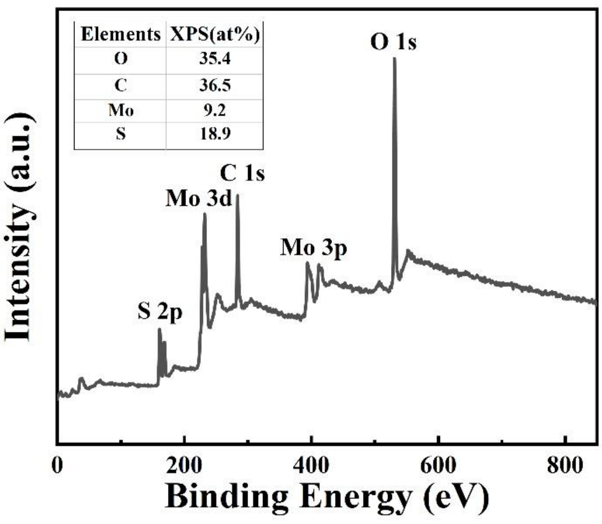

Figure S2. Survey spectra of MCM composite, insets show the actual content of O, C, Mo and S elements.

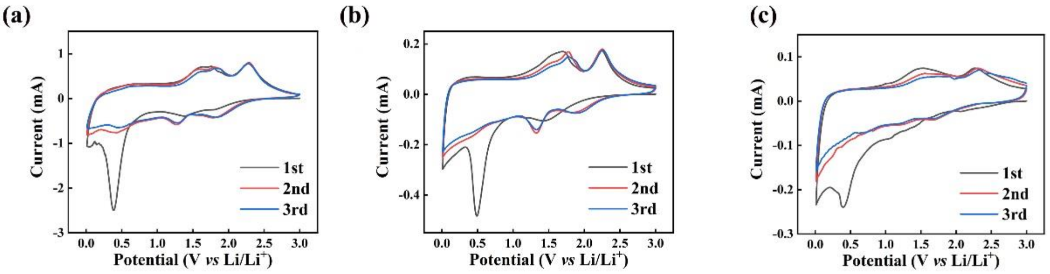

Figure S3. CV curves at 0.01 mV s^{-1} of pure MoS_2, MCS and MCL.

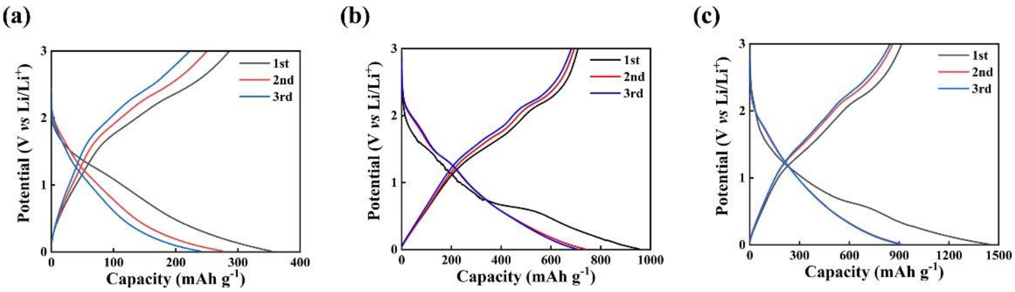

Figure S4. Charge-discharge profiles of pure MoS_2, MCS and MCL.

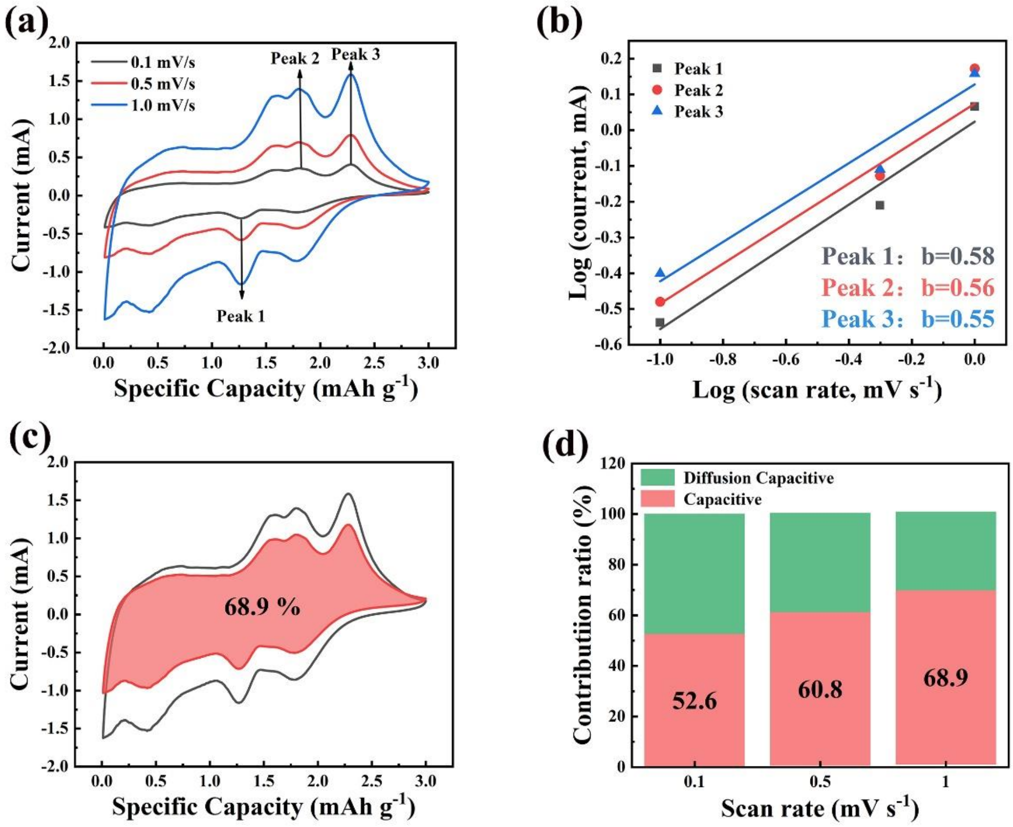

Figure S5. (**a**) CVs of pure MoS_2 electrode (0.1-1.0 V·s^{-1}), (**b**) Fitting line of log (v, mV/s)-log (I_{peak}, mA), (**c**) Capacitive contribution at 1.0 mV·s^{-1}, (**d**) Ratios of pseudocapacitive and diffusion controlled contributions at different sweep rates.
